# Theory of Mind as a Correlate of Bystanders’ Reasoning About Intergroup Bullying of Syrian Refugee Youth

**DOI:** 10.3389/fpsyg.2022.815639

**Published:** 2022-03-30

**Authors:** Seçil Gönültaş, Kelly Lynn Mulvey

**Affiliations:** ^1^Department of Psychology, University of Exeter, Exeter, United Kingdom; ^2^Department of Psychology, North Carolina State University, Raleigh, NC, United States

**Keywords:** intergroup bullying, bystander judgments, outgroup theory of mind, ingroup theory of mind, fairness, refugee status, discrimination, group functioning

## Abstract

The current study examined how ingroup and outgroup Theory of Mind (ToM) predicts children’s and adolescents’ reasoning for their acceptability judgments of intergroup bullying of Syrian refugee peers and group support of intergroup bullying. Participants included 587 Turkish middle (*n* = 372, *M*_*age*_ = 12.19, *SD* = 1.01; 208 girls) and high school (*n* = 215, *M*_*age*_ = 14.81, *SD* = 0.97; 142 girls) students. Participants read a bias-based bullying story with a Syrian refugee peer targeted by an ingroup Turkish peer. Then, participants rated the acceptability of bullying and group support of bullying and were presented with a reasoning question (Why?) after each acceptability question (bullying and group support of bullying). Reasoning codes included Fairness, Refugee Status/War, Prejudice and Discrimination, Harm, Prescriptive Norms, Group Functioning, and Relationship with the Bully. Participants’ ingroup and outgroup ToM abilities (measured using the Strange Stories) were evaluated as predictors of reasoning. Results documented that middle school students were more likely to attribute mental states to their ingroup members compared to outgroup members while high school students’ ToM performance did not differ across contexts. Further, the more unacceptable participants judged bullying to be, the more they reasoned about the bullying by referencing fairness, refugee status, discrimination, and harm. Results also documented that ingroup and outgroup ToM were positively related to attribution to fairness and participants’ usage of multiple reasoning judgments while only outgroup ToM was a significant predictor of reasoning around refugee status/war, discrimination, and prejudice. The findings provide implications for intervention programs that tackle intergroup bullying by examining bystanders’ social cognitive skills in a specific context.

## Introduction

Recent studies showed that Syrian refugee youth in Turkey are highly prone to experiencing intergroup school bullying rooted in racial discrimination and prejudice (Demir and Özgül, 2019; Çeri et al., 2021). Intergroup bullying refers to repeated aggressive behaviors and attitudes that harm someone within the context of a power imbalance because of a particular group membership (e.g., nationality, immigration/refugee background, religion, gender, sexual orientation, or disability, Palmer and Abbott, 2018). Considering the widespread and long-lasting effects of bullying on refugee youth (psychological well-being, physical health, educational attainment), it is critically important to identify the ways to promote anti-bullying intervention programs to foster inclusive schools. Although much research has focused on victims and bullies, bystanders, peers who witness bullying, are also central actors to stop bullying (Salmivalli et al., 2011). Thus, it is important to understand bystanders’ judgments and reasoning about intergroup bullying. As a critical social-cognitive skill, Theory of Mind (ToM), may be related to bystanders’ judgments of intergroup bullying as ToM may enhance one’s understanding of the prejudicial roots behind the bullying (Gönültaş and Mulvey, 2021a). However, the possible association between adolescents’ reasoning about intergroup bullying and ToM has not been explored yet. In the current study, we examined whether ToM predicts adolescents’ reasoning for their acceptability judgments of intergroup bullying of Syrian refugee peers and group support of intergroup bullying.

### Bystander Judgments About Intergroup Bullying From Social Reasoning Developmental Perspective

In the current study, we used the Social Reasoning Developmental (SRD) approach informed by the Social Domain Theory (SDT; Turiel, 1983) and Social Identity Development Theory (SIDT, Nesdale, 2004) to examine children’s and adolescents’ reasoning patterns. SDT posits that individuals reason about social decisions by considering three domains: the moral domain (issues of fairness, justice, and rights), the societal domain (customs, conventions, and traditions), and the psychological domain (related to personal choice and autonomy) (Smetana et al., 2014). Further, SDT suggests that children and adolescents evaluate social transgressions across domains of reasoning that may involve ToM (Smetana, 2006; Killen et al., 2011). We examined both ingroup and outgroup ToM as our hypothetical intergroup bullying scenario involves ingroup and outgroup characters.

SIDT suggests that children and adolescents develop intergroup attitudes toward outgroups in four phases: (1) not having a salient group membership, (2) group membership awareness, (3) ingroup preference, (4) ingroup bias, prejudice, and discrimination (Nesdale, 2004). According to SIDT, children’s and adolescents’ evaluations of bullying can be influenced by intergroup processes (e.g., group membership, prejudice, discrimination, and threat perception) if the bullying involves ingroup and outgroup members. For example, Ojala and Nesdale (2004) demonstrated that students aged between 10 and 13 evaluated bullying as more okay when the victim was an outgroup member who was perceived threat to ingroup members. Moreover, Jones et al. (2012) documented that Italian children aged 10–13 who were exposed to a cooperative group norm were more likely to feel negative emotions such as anger or regret following intergroup bullying. Thus, children and adolescents evaluate bullying by considering group-related factors and bring their social-cognitive awareness to these evaluations. By bringing SDT and SIDT approaches together, SRD approach proposes that children’s and adolescents’ judgments and evaluations in the context of intergroup social conflicts depend on both intergroup-related factors (e.g., group membership, prejudice, discrimination, and threat perception) and social cognitive skills (e.g., theory of mind, empathy). SRD approach also contends that children and adolescents often evaluate social conflicts as unacceptable by considering moral concerns but might support social conflicts due to group membership, group wellbeing, and functioning (e.g., Killen and Rutland, 2011; Killen et al., 2013; Hitti et al., 2014).

### Group Membership in the Context and Its Relation to Bystanders’ Judgments and Reasoning

Previous research drawing on SRD approach to intergroup bullying showed that group membership of the victim (whether the victim is an ingroup member or outgroup member) is related to bystander judgments and responses (Gönültaş and Mulvey, 2021a; Palmer et al., 2022). For example, Gönültaş and Mulvey (2021a) showed that non-immigrant adolescents were more likely to evaluate bullying as unacceptable when the victim was a non-immigrant peer compared to when the victim was an immigrant peer in the United States. Similarly, Palmer et al. (2022) found that Cypriot adolescents showed higher prosocial tendencies toward Cypriot victims than non-Cypriot victims in the context of social exclusion, while no differences were observed in non-Cypriot adolescents’ prosocial bystander responses. Relatedly, children and adolescents’ reasoning justifications can be dependent on the context and group membership of the victim. For example, Mazzone et al. (2018) documented that participants (aged between 11 and 15 years) differed in their answers when they were asked to think about the possible underlying reasons for the bullying across two scenarios (immigrant and non-immigrant victim). When the victim was an immigrant peer, participants were more likely to think that the reasons for the bullying can be cultural differences, coming from a different country, language, religion and feelings of fear toward immigrants even when the reason for bullying was not explicitly given. However, participants were more likely to think that the bullying could be related to victims’ personality characteristics and physical appearance when the victim was a non-immigrant peer (Mazzone et al., 2018).

### Theory of Mind in the Context and Its Relation to Bystanders’ Judgments and Reasoning

Theory of mind can be described as the ability to attribute and to predict subjective mental states of others including intentions, beliefs, desires, and emotions (ToM; Wellman et al., 2001; White et al., 2009). ToM is related to many social outcomes including prosocial behaviors across childhood and adolescence (Razza and Blair, 2009; Imuta et al., 2016). As an important social cognitive ability, ToM may be one of the factors that helps children and adolescents to understand and reason about underlying motives behind different types of bullying (e.g., intragroup and intergroup). Although ToM has been broadly defined by the successful performance in false-belief tasks around age three, ToM competence involves a long and nuanced developmental path (Peterson and Wellman, 2019). Wellman and Liu (2004) developed a ToM battery to be able to comprehensively evaluate several aspects of ToM across preschool and childhood, but development of these skills does not cease during childhood. Moving through adolescence, ToM abilities continue to grow across this period including understanding sarcasm, irony, white lies, and deception (Dumontheil et al., 2010; Peterson and Wellman, 2019). The Strange Stories is one of the widely used tasks to measure advanced ToM skills (White et al., 2009). This measure involves several stories tapping into different sub-domains of ToM including the understanding of mental states in contexts involving white lies, persuasion, and misunderstanding. Thus, ToM is a complex social-cognitive skill that has been measured with a range of tasks requiring individuals to infer others’ mental states. What has not yet been demonstrated, however, is if this social-cognitive competency is related to individuals’ reasoning about situations that may rely on mental-state knowledge, such as intergroup bullying situations.

SRD model also argues that social-cognitive skills (e.g., ToM) are related to children’s and adolescents’ reasoning as these skills help them to recognize the multifaceted nature of social conflicts (Rutland and Killen, 2015). Thus, it is likely that children and adolescents with higher social-cognitive skills can consider different aspects of a social conflict and weigh multiple considerations including the moral domain (issues of welfare, justice, and rights), the societal domain (concerns group functioning and social norms) and the psychological domain (concerns one’s choice over private matters) (Turiel, 1983; Rutland and Killen, 2015).

ToM also fosters more complex moral reasoning while evaluating intentional and unintentional social transgressions (Killen et al., 2011; D’Esterre et al., 2019; Baker et al., 2021). Previous research has demonstrated that ToM is related to active bystander challenging behavior in generalized bullying (Gini, 2006; Caravita et al., 2010). ToM predicted higher defending behaviors against intragroup bullying through increased social competence (Metallidou et al., 2018). More recently, studies also examined the possible role of ToM in bystander responses to intergroup bullying of immigrant youth (Gönültaş and Mulvey, 2021a,b). Accordingly, adolescents’ ToM abilities predicted a higher likelihood of bystander active responses to both bullying and following retaliatory acts in intergroup contexts (e.g., when an immigrant peer is victimized or seeks retaliation, Gönültaş and Mulvey, 2021a,b).

In all these studies, ToM was evaluated as a generalized ability. However, more recently, studies showed that children and adolescents can be selective while using their efforts to attribute mental states to their ingroup and outgroup members (McLoughlin and Over, 2017; McLoughlin et al., 2018; Gönültaş et al., 2020). For example, across two studies children attributed less humanness and fewer mental states (e.g., believing, pretending, and deciding) to agents from different groups (geographically based groups and gender-based groups) and they were more likely to use their ToM skills for their ingroup members than for outgroup members (McLoughlin and Over, 2017; McLoughlin et al., 2018). Overall, these studies suggest that children’s ingroup ToM and outgroup ToM performance differ.

Two studies have been examined outgroup ToM in Turkish adolescents (Gönültaş et al., 2020) and young adults (Ekerim-Akbulut et al., 2020) to examine their mental state attribution to Syrian refugee individuals. Gönültaş et al. (2020) demonstrated that Turkish adolescents were more likely to attribute mental states to Turkish story characters (ingroup ToM) compared to Syrian refugee characters (outgroup ToM) in a study using the Strange Stories task. They also found that discrimination and threat perception toward refuges were negatively related to adolescents’ outgroup ToM performance. Similarly, Ekerim-Akbulut et al. (2020) also showed that Turkish college students’ ToM abilities differ based on participants’ perceived similarity with the refugees by using the Strange Stories task. More specifically, participants who reported lower perceived similarity with the targeted outgroups (refugees) showed worse performance in attributing mental states to Syrian refugee individuals compared to Turkish individuals.

Based on previous studies, it is likely that the group membership of the target might matter for both children’s and adolescents’ reasoning/judgments and their ToM performance in intergroup contexts. Further, ingroup and outgroup ToM might shape children’s and adolescents’ reasoning about intergroup social conflicts, which involve ingroup and outgroup members. Although separate research lines on bystanders’ judgments and reasoning in intergroup bullying and ToM in intergroup context are gaining attention, still little is known about how the intersection of these lines of research might help us to have a more context-sensitive understanding of the relationship between ToM and reasoning in an intergroup context.

### High-Tension Intergroup Context: Intergroup Bullying of Syrian Refugees in Turkey

The refugee crisis is a global issue that impacts many societies across the world. Turkey, as one of those countries, has received more than three million seven hundred thousand refugees from the start of the crisis in Syria in 2011 through 2021 (United Nations High Commissioner for Refugees [UNHCR], 2021). Syrian children and adolescents constitute a great percentage of the Syrian refugee population in Turkey (United Nations High Commissioner for Refugees [UNHCR], 2021). Thus, it is highly likely that non-refugee children and adolescents can have opportunities for contact with Syrian peers. Relatedly, the intergroup interactions between Turkish and Syrian adolescents are high, especially in public schools (Gönültaş et al., 2020; Içduygu and Nimer, 2020). Turkey also has a quite high prevalence rate of bullying (24%) in schools (Programme for International Student Assessment, 2018). Although these reports do not provide information about the ethnic background of victims or the reason for the bullying, recent studies showed that Syrian refugee youth are highly prone to experiencing intergroup school bullying rooted in racial discrimination and prejudice. For example, Karaman (2021) examined the rates of bullying among Syrian and Turkish students and found that Syrian students reported higher rates of bullying victimization compared to their Turkish peers. A qualitative study by Demir and Özgül (2019) also demonstrated that Syrian refugee students were at an increased risk of being bullied due to discrimination, language barriers, and cultural differences. One way to reduce intergroup bullying is promoting active upstanding behavior of non-refugee peers who witness the intergroup bullying of their refugee peers. Bystander peers can serve as central actors to offset both the occurrence and effects of bullying (Salmivalli et al., 2011) when they show defending behaviors (e.g., challenging the bully and supporting the victim). However, to date, little research has focused on how non-refugee adolescents evaluate intergroup bullying of their refugee peers and how ToM abilities for ingroup and outgroup members might be related to their reasoning.

### Present Study

This study is a part of a larger project that investigated the bystander judgments and bystander responses to generalized and intergroup bullying and inclusivity judgments (Gönültaş et al., 2021). The purpose of the present study was to examine how ingroup and outgroup ToM can be related to bystanders’ judgments and reasoning to intergroup bullying of refugee peers. Examining how children and adolescents evaluate and reason about the intergroup bullying of refugees provides novel information regarding the contexts in which they challenge it and seek to stop it from occurring in the future. Further, it is also important to identify factors involved in children’s and adolescents’ reasoning to inform intervention programs. However, our knowledge is limited in terms of how social-cognitive factors can be related to adolescents’ judgments and reasoning as bystanders when they are evaluating intergroup bullying. To address this, we examined to what extent adolescents’ bystander judgments and reasoning might be related to their ingroup and outgroup ToM abilities.

We conducted our research with middle and high school students as previous studies showed some age-related patterns. For example, middle school students were less likely to see bullying as acceptable and were less likely to show active responses compared to high school students (e.g., Mulvey et al., 2019). Further, the SRD approach also contends that children’s decisions, judgments, and reasoning about social conflicts increasingly involve intergroup-related factors with age (Killen and Rutland, 2011).

**Hypotheses related to differences in ingroup–outgroup ToM and acceptability judgments based on gender and age:**

1. Based on earlier studies (McLoughlin and Over, 2017; McLoughlin et al., 2018; Gönültaş et al., 2020), we expected that participants would be more likely to attribute mental states to their ingroup members compared to their outgroup members.
2. Previous studies showed that females and younger adolescents were more likely to evaluate bullying as unacceptable and were more likely to show active bystander responses to bullying (Mulvey et al., 2019). Thus, based on the previous studies we hypothesized that middle school students and female students would be likely to evaluate intergroup bullying and group support as less acceptable compared to high school and male students.

**Main Hypotheses related to the relationship between ingroup and outgroup ToM, acceptability judgments, and reasoning**:

3. Participants’ ingroup and outgroup ToM would positively predict participants’ attribution to “fairness,” “welfare,” “prejudice and discrimination, refugee status” in their acceptability judgments about the intergroup bullying and group support of intergroup bullying. The categories were considered under the moral domain based on the previous studies (e.g., Ruck et al., 2015). We also expected that participants’ outgroup ToM would more strongly predict the reasoning related to “fairness,” “welfare,” “prejudice and discrimination, refugee status” compared to ingroup ToM as outgroup ToM involves the understanding mental states of outgroup members.
4. We expected that the more unacceptable participants judged bullying and group support to be, the more they would reason about the bullying by referencing “fairness,” “welfare,” “prejudice and discrimination, refugee status.” Further, as participants evaluate intergroup bullying and group support as more acceptable, they would be more likely to attribute to “prescriptive norms,” “group functioning,” and “relationship with the bully.”
5. Participants with higher ingroup and outgroup ToM would be more likely to attribute more than one justification in their reasoning than those with lower ToM.

## Materials and Methods

### Participants

Data was collected from 587 Turkish adolescents in high (*n* = 215, *M*_*age*_ = 14.81, *SD* = 0.97; 142 girls) and middle (*n* = 372, *M*_*age*_ = 12.19, *SD* = 1.01; 208 girls) school in Istanbul, Turkey. Istanbul hosts more than half a million Syrian refugees (Directorate-General for Migration Management, 2021). We collected data from eight schools in four different districts: two districts with relatively a higher number of Syrian refugees and two districts with a relatively lower number of Syrian refugees. Syrian youth were not recruited as there were measures related to attitudes toward Syrian refugees in Turkey. A power analysis using G*Power showed that a sample size of at least 382 participants would be needed with the desired statistical power at 0.95, and an alpha of 0.05 (Faul et al., 2009) for the logistic regression analyses.

### Procedure

Ethical approvals were obtained from two universities (in the United States and in Turkey). Students were recruited by sending invitation letters and consent forms to parents through their schools. All students with parental consent who assented to participate were included in the study. Participants completed the survey in a paper-based format in their schools. All measures were presented in Turkish. We collected the data between December 9, 2019, and January 10, 2020. Students were given small gifts (pencils, etc.) as compensation for their participation.

### Measures

#### Intergroup Bullying Scenario and Acceptability Judgments

Participants read the following hypothetical scenario in which a Syrian refugee peer is bullied because of his/her refugee status (intergroup bullying). The story was created based on earlier research (Gönültaş and Mulvey, 2019; Mulvey et al., 2019) and was adapted and translated for this study using forward-translation and back-translation methods. Common Turkish and Syrian names were used in the story and the story was gender-matched to participants.

*“Your group enjoys telling each other jokes about lots of things, including about different groups of people. Now, imagine that the school day has not yet started, and you are hanging out with your group of friends in the hallway. There are no teachers around yet. Barış (ingroup bully), who is one of the kids in your group of friends, shouts out rude words against Syrian people. Meanwhile, Joram (outgroup victim) appears. Joram is originally from Syria but now lives in Turkey. When Barış realizes Joram is around, he purposely shouts out a rude word at Joram because Joram is from Syria as he did in the previous days.”*

#### Acceptability Judgment for the Intergroup Bullying

Participants rated their acceptability judgment for the intergroup bullying with the following question “How okay or not okay is it that Barış acts this way?” on a six-point Likert scale ranging from 1 (*really not okay*) to 6 (*really okay*).

#### Acceptability Judgment for the Group Support of the Intergroup Bullying

Then participants were presented with the following scenario indicating group support for the bully *“Because your group enjoys telling jokes about lots of things, including about different groups of people, your group finds what Barış did funny and starts to laugh to support him.”* Then, they were asked to rate the acceptability of group support with the following questions “How okay or not okay is your group for agreeing that shouting rude words to someone from a different country is funny?” on a six-point Likert scale ranging from 1 (*really not okay*) to 6 (*really okay*). The explanations for the character names in parentheses (“ingroup bully and outgroup victim”) were not provided in the actual surveys.

#### Reasoning for the Acceptability Judgments

After participants completed their acceptability judgments for the intergroup bullying and their group support for intergroup bullying, they were presented with a reasoning question (“Why?”) and they provided open-ended responses. Participants’ responses were coded based on a coding system developed from the previous literature on individuals’ conceptions of moral judgments and SDT theory (Killen et al., 2013). The coding framework based on SDT has been also used in Turkish (Gönül and Acar, 2018) with similar concepts including fairness, harm, etc.

For the analyses, we only used the codes that frequency percentages were more than 10%. To achieve 10%, we merged “Refugee Status/War” and “Prejudice and Discrimination” categories as a single category (“Discrimination, Prejudice, Refugee Status and War”) for both outcomes. For the reasoning of the acceptability of intergroup bullying analyses four codes have emerged: (1) Fairness, (2) Discrimination, Prejudice, Refugee Status and War, (3) Harm and (4) Prescriptive Norms (please see Table 1 for frequencies and examples for each code). For the reasoning of the acceptability of group support to the intergroup bullying four codes have emerged: (1) Discrimination, Prejudice, Refugee Status and War, (2) Harm, (3) Group Functioning and (4) Relationship with the Bully (please see Table 1 for frequencies and examples for each code). Interrater reliability between coders was assessed based on about 25% of the interviews, with very good reliability, Cohen’s κ = 0.89. Further, we also wanted to examine whether participants’ ToM abilities were related to their attribution to multiple categories. Thus, participants were given “Yes/1” for the categories that they referred to and “No/0” for the categories that they did not attribute. When participants referred to multiple categories, they were given “Yes/1” for each code used (up to three codes per response were recorded).

**TABLE 1:** Examples and percentages of reasoning for acceptability judgments.

Judgment	Reasoning (percentages)	Example
Acceptability judgments to intergroup bullying	Fairness (12.1%)	It is not fair to bully anyone for any reason
	Discrimination, Prejudice, Refugee Status and War (50.2%)	We shouldn’t treat like this her just because she is a refugee from Syrian/It’s racist and discriminatory
	Harm (20.4%)	It will hurt his feelings
	Prescriptive Norms (13.3%)	Because you are not supposed to bully
Acceptability judgments for group support of intergroup bullying	Discrimination, Prejudice, Refugee Status and War (14.3%)	The situation can be worse if the group supports and everyone in the school may hate from Syrian refugees.
	Harm (21.2%)	The girl is already sad. And if they laugh too, she can get more upset.
	Group Functioning (13.5%)	I don’t want to ruin the unity of the group over a little joke.
	Relationship with the Bully (13.1%)	I and my friends support Barış because he is our friend. I don’t want to lose my friend because of a person that I do not know.

*Example responses were translated from Turkish to English.*

#### Theory of Mind

Participants’ ToM for the targeted outgroup and ingroup was measured using a modification of the Strange Stories measure (White et al., 2009; Devine and Hughes, 2016; Gönültaş et al., 2020). We adapted two mind-reading stories (white lie and persuasion) by referencing Syrian individuals and the other two mind-reading stories (white lie and misunderstanding) by referencing Turkish individuals. All participants were presented with both ingroup and outgroup ToM (within-subject effect). Thus, different stories were used to avoid practice effects. After each story, participants were asked to answer a question that requires understanding the mental state of the characters in the story. Participants’ answers were coded by using the following criteria *2* = *correct answer with mental state attribution; 1* = *correct information without attributing mental states and 0* = *false answer*. Interrater reliability (based on 25% of responses) was Cohen’s κ = 0.96. Participants’ performance was calculated by summing scores from two stories (ranged between 0 and 4) for both ingroup ToM and outgroup ToM. The Turkish version of this measure has been previously used by earlier studies (Ekerim-Akbulut et al., 2020; Gönültaş et al., 2020).

### Data Analysis Plan

First, bivariate correlation analyses were conducted to examine the possible correlation between gender, age, ingroup ToM, outgroup ToM, acceptability judgments to intergroup bullying, and group support. Second, to examine differences between ingroup and outgroup ToM based on gender (female/male) and school (middle/high) a mixed ANOVA was conducted. Then, an ANCOVA was conducted to compare participants’ acceptability judgments for intergroup bullying with their judgments of the acceptability for group support by school and gender controlling for ToM. Lastly, to examine the relationship between participants’ reasoning for acceptability judgments for intergroup bullying and group support and their ingroup and outgroup ToM abilities (continuous), separate Logistic Regressions were conducted. Age, gender, and participants’ acceptability judgments were also added to the analyses as possible predictors.

## Results

### Preliminary Analyses

#### Correlations

Bivariate Pearson correlations showed that there was a statistically significant positive correlation between ingroup and outgroup ToM (*r* = 0.33, *p* < 0.001). Further, ingroup ToM was negatively associated to acceptability judgments of intergroup bullying (*r* = −0.11, *p* = 0.012) and group support (*r* = −0.11, *p* = 0.011) while outgroup ToM was only negatively correlated with acceptability of group support (*r* = −0.10, *p* = 0.021) (please see Table 2 for correlations).

**TABLE 2:** Correlations among study variables.

	1	2	3	4	5
Gender (0 = female, 1 = male)	−				
Age (0 = middle, 1 = high)	−0.10*	−			
Ingroup ToM	–0.04	–0.02	−		
Outgroup ToM	−0.21***	0.16***	0.33***	−	
Acceptability of intergroup bullying	0.08*	0.06	−0.11*	–0.07	−
Acceptability of group support of intergroup bullying	0.08*	0.04	−0.11*	−0.10*	0.62***

*p < 0.05, ***p < 0.001.

#### Differences Among Ingroup and Outgroup Theory of Mind

**FIGURE 1:**
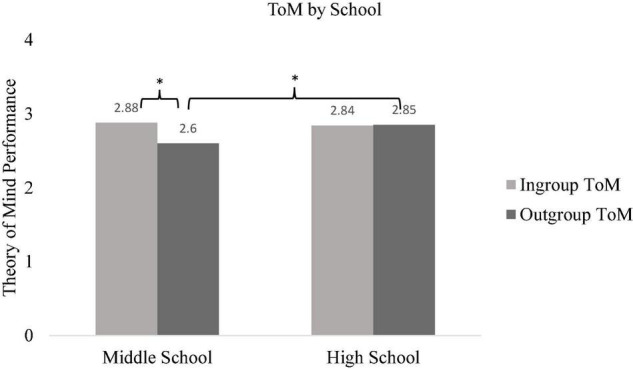
Participants’ ingroup and outgroup ToM by school. *p < 0.05.

**FIGURE 2:**
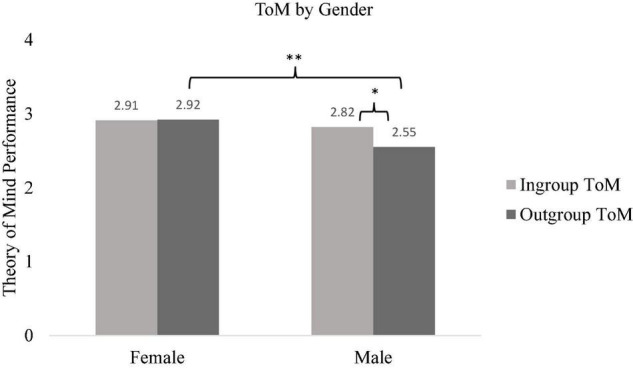
Participants’ ingroup and outgroup ToM by gender. *p < 0.05, ^**^p < 0.01.

A 2 (ToM: ingroup and outgroup) × 2 (gender: male and female) × school (high school and middle school) repeated measures ANOVA was conducted, with ToM as within-subject and gender and school as between-subject factors to test H1. Results documented a main effect of ToM indicating that participants’ ingroup ToM was higher compared to their outgroup ToM [*F*(1,534) = 7.36, *p* = 0.007, $\eta_{\text{p}}^{2}$ = 0.014]. Further, a significant two-way interaction between ToM and school was observed [*F*(1,534) = 9.13, *p* = 0.003, $\eta_{\text{p}}^{2}$ = 0.017]. Pairwise comparison (with Bonferroni corrections) showed that middle school students were more likely to attribute mental states to their ingroup members compared to outgroup members [*F*(1,534) = 23.99, *p* < 0.001, $\eta_{\text{p}}^{2}$ = 0.043]. However, high school students’ ToM performance did not differ between ingroup and outgroup members [*F*(1,534) = 0.04, *p* = 0.449, $\eta_{\text{p}}^{2}$ = 0.000]. Further, high school students’ outgroup ToM was higher compared to middle school students’ outgroup ToM [*F*(1,534) = 8.12, *p* = 0.005, $\eta_{\text{p}}^{2}$ = 0.015] while ingroup ToM did not differ between middle and high school students [*F*(1,534) = 0.30, *p* = 0.587, $\eta_{\text{p}}^{2}$ = 0.001] (see Figure 1). Lastly, results showed a significant interaction between ToM and gender [*F*(1,534) = 8.78, *p* = 0.003, $\eta_{\text{p}}^{2}$ = 0.016]. Accordingly, male participants were more likely to attribute mental states to their ingroup members compared to outgroup members [*F*(1,534) = 18.43, *p* = 0.001, $\eta_{\text{p}}^{2}$ = 0.033], however, females’ ingroup and outgroup ToM did not differ [*F*(1,534) = 1.19, *p* = 0.276, $\eta_{\text{p}}^{2}$ = 0.002] (see Figure 2).

### Acceptability Judgments for Intergroup Bullying and Group Support

A 2 (acceptability of intergroup bullying and acceptability of group support to intergroup bullying) × 2 (gender: male and female) × school (high school and middle school) repeated measures ANCOVA was conducted, with acceptability judgments as within-subject and gender and school as between-subject factors (to test H2). Ingroup and outgroup ToM were included as covariates in the analysis as they were significantly correlated with the acceptability judgments. Results did not document a main effect of acceptability judgments indicating that participant did not differ in their judgments between acceptability of intergroup bullying (*M* = 1.64, *SD* = 0.05) and group support of bullying (*M* = 1.63, *SD* = 0.05), *F* (1, 527) = 0.73, *p* = 0.787, $\eta_{\text{p}}^{2}$ = 0.000. Only the main effect of gender was significant [*F*(1,527) = 4.76, *p* = 0.030, $\eta_{\text{p}}^{2}$ = 0.009]. Overall (across intergroup bullying and group support), females’ acceptability judgments (*M* = 1.53, *SD* = 0.06) were lower compared to males (*M* = 1.74, *SD* = 0.07). None of two way or three-way interactions were significant. In general, participants judged bullying and group support for bullying as unacceptable (all the means below midpoint 3).

### Main Analyses

Initial frequencies demonstrated that different categories have emerged for the acceptability of intergroup bullying. Thus, for the reasoning about the acceptability of intergroup bullying analyses, we conducted four logistic regression analyses by using the following categories as our outcome variables: (1) Fairness, (2) Discrimination, Prejudice, Refugee Status and War, (3) Harm, and (4) Prescriptive Norms. For the reasoning about the acceptability of group support of intergroup bullying analyses, we conducted four logistic regression analyses by using the following categories as our outcome variables: (1) Discrimination, Prejudice, Refugee Status and War, (2) Harm, (3) Group Functioning, and (4) Relationship with the Bully. Lastly, we conducted two additional logistic regression analyses to see whether participants’ ToM abilities (ingroup and outgroup) were related to their reference to multiple categories in their reasoning for their acceptability judgments for intergroup bullying and group support to intergroup bullying. Overall, five separate regressions were conducted for each outcome variable.

### Reasoning Analyses for Acceptability Judgments for Intergroup Bullying

With regard to fairness reasoning, the overall model indicates a significant fit [χ^2^(4, *N* = 463) = 35.68, Nagelkerke *R*^2^ = 0.10, *p* < 0.001] and the variables included made significant contributions to the model (H3 and H4). Results showed that the more unacceptable participants judged bullying to be, the greater the odds that they reasoned about the bullying by referencing fairness [β = −0.29, χ^2^(1) = 8.53, *p* = 0.003, *Exp(B)* = 0.768, 95% CI [0.61, 0.91]]. Similarly, participants’ ingroup ToM [β = 0.34, χ^2^(1) = 6.73, *p* = 0.009, *Exp(B)* = 1.41, 95% CI [1.08, 1.83]] and outgroup ToM abilities [β = 0.32, χ^2^(1) = 7.32, *p* = 0.007, *Exp(B)* = 1.37, 95% CI [1.09, 1.72]] were positively related to participants’ fairness justifications. School and gender were not significant predictors of participants’ fairness justifications (see Table 3).

**TABLE 3:** Binary logistic regression analyses for reasoning of acceptability judgments to intergroup bullying.

	Fairness					Discrimination, Prejudice, Refugee Status and War					Harm				
	*B*	*SE*	*Wald*	*p*	*Exp(B)*	*B*	*SE*	*Wald*	*p*	*Exp(B)*	*B*	*SE*	*Wald*	*p*	*Exp(B)*
School	−0.06	0.06	0.96	0.327	0.94	−0.09	0.06	2.07	0.150	0.91	0.00	0.06	0.00	0.980	1.00
Gender	−0.10	0.20	0.25	0.616	0.90	−0.47	0.21	5.28	0.022	0.62	0.20	0.20	1.02	0.313	1.22
Acceptability	−0.29	0.10	8.53	0.003	0.75	−0.45	0.12	14.47	0.000	0.64	−0.34	0.10	11.09	0.001	0.71
Outgroup ToM	0.32	0.12	7.32	0.007	1.37	0.35	0.12	8.30	0.004	1.42	0.00	0.11	0.00	0.983	1.00
Ingroup ToM	0.35	0.13	6.74	0.009	1.41	0.25	0.14	3.51	0.061	1.29	0.02	0.12	0.04	0.846	1.02
*Chi square*	35.68					50.66					14.34				
*Model sig.*	<0.001					<0.001					0.014				
*Nagelkerke R* ^2^	0.10					0.14					0.04				

For discrimination, prejudice and refugee status/war, the null model significantly improved with all the predictors in the model (age, gender, acceptability judgments, ToM), χ^2^(4, *N* = 463) = 50.66, Nagelkerke *R*^2^ = 0.14, *p* < 0.001 (H3 and H4). Accordingly, the effect of gender was significant, documenting that female participants were more likely to refer to refugee status/war than were male participants [β = −0.47, χ^2^(1) = 5.28, *p* = 0.022, *Exp(B)* = 0.62, 95% CI [0.42, 0.95]]. Results also documented that the more participants evaluated intergroup bullying as acceptable, the less likely they were to refer to refugee status/war [β = −0.45, χ^2^(1) = 14.47, *p* < 0.001, *Exp(B)* = 0.64, 95% CI [0.50, 0.80]]. Further, participants’ higher outgroup ToM score was a significant positive predictor of participants’ attribution to refugee status/war reasoning justification for their evaluation of intergroup bullying [β = 0.35, χ^2^(1) = 8.30, *p* = 0.004, *Exp(B)* = 1.42, 95% CI [1.12, 1.81]]. Neither school nor ingroup ToM were significant correlates of participants’ reference to refugee status/war (see Table 3).

The overall model for harm reasoning was also significant [χ^2^(4, *N* = 463) = 14.34, Nagelkerke *R*^2^ = 0.05, *p* = 0.014]. Neither gender, school nor ToM were found as significant predictors (H3 and H4). The only significant predictor was acceptability judgments indicating that the more unacceptable participants judged bullying to be, the greater the odds that they reasoned about the bullying by referencing harm [β = −0.34, χ^2^(1) = 11.10, *p* = 0.001, *Exp(B)* = 0.711, 95% CI [0.58, 0.86]] (see Table 3).

The overall model for prescriptive norm reasoning was also significant [χ^2^(4, *N* = 463) = 13.93, Nagelkerke *R*^2^ = 0.05, *p* = 0.016] (H3 and H4). The only significant predictor was acceptability judgments. More specifically, increasing acceptability was associated with a decreased likelihood of attributing prescriptive norms [β = −0.60, χ^2^(1) = 6.23, *p* = 0.013, *Exp(B)* = 0.549, 95% CI [0.34, 0.87]]. Neither gender, school outgroup ToM nor ingroup ToM were found as significant predictors (see Table 4).

**TABLE 4:** Binary logistic regression analyses for reasoning of acceptability judgments to intergroup bullying.

	Prescriptive Norms					Multiple Reasoning Attribution				
	*B*	*SE*	*Wald*	*p*	*Exp(B)*	*B*	*SE*	*Wald*	*p*	*Exp(B)*
School	0.07	0.08	0.75	0.388	1.08	−0.01	0.07	0.05	0.824	0.99
Gender	−0.29	0.29	1.00	0.318	0.75	−0.19	0.21	0.78	0.377	0.83
Group support	−0.60	0.24	6.23	0.013	0.55	−0.07	0.11	0.36	0.547	0.94
Outgroup ToM	−0.17	0.15	1.16	0.281	0.85	0.25	0.12	4.61	0.032	1.29
Ingroup ToM	0.22	0.18	1.44	0.230	1.24	0.30	0.14	4.78	0.029	1.35
*Chi square*	13.93					16.32				
*Model sig.*	0.016					0.006				
*Nagelkerke R* ^2^	0.05					0.05				

Lastly, our logistic regression to understand predictors of participants’ likelihood of using multiple types of reasoning for the acceptability judgments of intergroup bullying showed that both outgroup ToM [β = 0.25, χ^2^(1) = 4.61, *p* = 0.032, *Exp(B)* = 1.29, 95% CI [1.02, 1.62]] and ingroup ToM [β = 0.30, χ^2^(1) = 4.680, *p* = 0.029, *Exp(B)* = 1.35, 95% CI [1.03, 1.76]] positively predict the usage of multiple categories in participants’ reasoning (H5). Neither gender, age, acceptability judgment nor ingroup ToM were found as significant predictors (see Table 4).

### Reasoning Analyses for Group Support of Intergroup Bullying

The logistic regression model for prejudice, discrimination and refugee status/war reasoning justification was not statistically significant [χ^2^(5, *N* = 463) = 10.75, Nagelkerke *R*^2^ = 0.04, *p* = 0.057]. None of the predictors were significant (see Table 5) (H3 and H4).

**TABLE 5:** Binary logistic regression analyses for reasoning of group support to intergroup bullying.

	Discrimination, Prejudice, Refugee Status and War					Harm					Group Functioning				
	*B*	*SE*	*Wald*	*p*	*Exp(B)*	*B*	*SE*	*Wald*	*p*	*Exp(B)*	*B*	*SE*	*Wald*	*p*	*Exp(B)*
School	0.03	0.08	0.13	0.717	1.03	0.04	0.07	0.38	0.539	1.05	−0.09	0.09	1.00	0.317	0.92
Gender	−0.52	0.30	3.00	0.083	0.59	0.46	0.23	3.85	0.050	1.58	−0.35	0.29	1.43	0.231	0.71
Group support	−0.23	0.17	1.86	0.173	0.80	−0.32	0.14	4.99	0.025	0.73	0.16	0.12	1.77	0.183	1.17
Outgroup ToM	0.30	0.17	2.94	0.086	1.35	0.09	0.13	0.43	0.510	1.09	−0.11	0.15	0.56	0.456	0.89
Ingroup ToM	−0.06	0.18	0.10	0.749	0.94	0.16	0.15	1.10	0.294	1.17	0.05	0.17	0.08	0.780	1.05
*Chi square*	10.75					11.25					4.37				
*Model sig.*	0.057					0.047					0.498				
*Nagelkerke R* ^2^	0.04					0.04					0.02				

With regard to harm reasoning about the acceptability of group support for intergroup bullying, the overall model was significant [χ^2^(5, *N* = 463) = 11.25, Nagelkerke *R*^2^ = 0.04, *p* = 0.047] (H3, H5 and H6). According to the last step in the model, male participants [β = 0.46, χ^2^(1) = 3.84, *p* = 0.049, *Exp(B)* = 1.58, 95% CI [1.00, 2.51]] and participants who evaluated group support of intergroup bullying as more acceptable [β = −0.32, χ^2^(1) = 4.99, *p* = 0.025, *Exp(B)* = 0.729, 95% CI [0.55, 0.96]] were less likely to attribute harm. Ingroup and outgroup ToM abilities were not significant predictors of participants’ harm reasoning to acceptability judgments for group support to intergroup bullying (see Table 5).

The overall model for group functioning was not significant [χ^2^(5, *N* = 463) = 4.36, Nagelkerke *R*^2^ = 0.02, *p* = 0.498] (H3 and H4). None of the predictors were significant (see Table 5).

Similarly, model yields non-significant results for the reasoning about the relationship with the bully [χ^2^(5, *N* = 463) = 5.32, Nagelkerke *R*^2^ = 0.02, *p* = 0.378] (see Table 6) (H3 and H4).

**TABLE 6:** Binary logistic regression analyses for reasoning of group support to intergroup bullying.

	Relationship with the Bully					Multiple Reasoning Attribution				
	*B*	*SE*	*Wald*	*p*	*Exp(B)*	*B*	*SE*	*Wald*	*p*	*Exp(B)*
School	−0.11	0.09	1.53	0.216	0.90	0.18	0.07	6.53	0.011	1.20
Gender	0.29	0.28	1.11	0.293	1.34	−0.11	0.22	0.28	0.599	0.89
Acceptability	−0.23	0.16	2.01	0.156	0.80	−0.14	0.13	1.12	0.291	0.87
Outgroup ToM	−0.01	0.15	0.00	0.960	0.99	0.07	0.12	0.39	0.533	1.08
Ingroup ToM	0.03	0.17	0.03	0.862	1.03	0.21	0.14	2.29	0.130	1.23
*Chi square*	5.32					10.83				
*Model sig.*	0.378					0.055				
*Nagelkerke R* ^2^	0.02					0.04				

Lastly, our logistic regression to understand predictors of participants’ likelihood of using multiple categories (yes/no) for the acceptability judgments for group support to intergroup bullying showed that the overall model fit was not significant [χ^2^(5, *N* = 463) = 10.83, Nagelkerke *R*^2^ = 0.04, *p* = 0.055] (H5). However, the results documented that high school students were more likely to refer to more than one domain compared to middle school students [β = 0.18, χ^2^(1) = 6.53, *p* = 0.011, *Exp(B)* = 1.19, 95% CI [1.04, 1.38]]. None of the other predictors were significant (see Table 6).

## Discussion

The extant body of research demonstrates the possible role of ToM in bystander judgments and reasoning; however, this relationship has not been explored by evaluating both ToM and bystander responses in intergroup contexts in concert. The current study examined how participants’ ingroup and outgroup ToM relate to their different types of reasoning when evaluating intergroup bullying and group support to intergroup bullying. The novel findings of our study demonstrated that ingroup and outgroup ToM were related to participants’ reasoning about their evaluation of intergroup bullying but not associated with their reasoning about group support to intergroup bullying. Further, only outgroup ToM predicted participants’ references to intergroup-related themes (e.g., discrimination, prejudice, refugee status/war).

In line with previous studies (Gönültaş et al., 2020), our study also showed that middle school students were more likely to attribute mental states to their ingroup members (Turkish story characters) compared to outgroup members (Syrian refugee story characters). However, high school students’ ingroup ToM and outgroup ToM performance did not differ from each other (H1 was partially supported). Earlier research examined this phenomenon in early childhood (McLoughlin and Over, 2017; McLoughlin et al., 2018), middle childhood (Gönültaş et al., 2020), and young adulthood (Perez-Zapata et al., 2016; Ekerim-Akbulut et al., 2020). It is likely that adolescents may have more opportunities for contact with Syrian refugees in their school environments compared to children and adults which may lead to an increase in perceived similarity. Further, it might be also that high school students have more knowledge about different social groups in the society compared to middle school students (Levy and Killen, 2010), leading to improved abilities to infer mental states about outgroup peers. Further, Gönültaş et al. (2020) found that perceived threat perception toward Syrian refugees was negatively related to middle school students’ outgroup ToM performance. It is likely that middle school students have a relatively higher threat perception toward Syrian refugees compared to high school students leading them to differentiate in their ToM performance across ingroup and outgroup members. The possible factors that might be related to the non-significant differences between ingroup and outgroup ToM in older adolescents should be examined further by using different ToM tasks as well.

Contrary to our hypothesis, we did not find any school (middle/high) or gender-related differences in participants’ acceptability judgments to intergroup bullying and group support (H2 was not supported). Thus far, similar age and gender-related patterns in bystanders’ judgments and responses were observed in generalized bullying that does not involve any intergroup-related processes (Mulvey et al., 2019; Gönültaş et al., 2019). However, there are mixed results about bystander responses in intergroup context documenting either no difference or reverse patterns (e.g., Yüksel et al., 2021). Thus, there is a need for further understanding of how different factors might be related to different age and gender patterns in bystander responses to different types of bullying.

Our novel findings suggested that ingroup ToM (ToM in a generalized context) positively predicted participants’ attribution to fairness in judging the acceptability of intergroup bullying. This is in line with previous studies documenting the relationship between generalized ToM and moral judgments in intergroup context (e.g., Burkholder et al., 2019; Gönültaş and Mulvey, 2021a). Considering earlier studies on the role of ToM for social relationships, it is plausible to conclude that this relation is mostly studied in a generalized context and less attention is paid to outgroup ToM. To our knowledge, for the first time, we have examined outgroup ToM and its relationship with the participants’ reasoning in addition to ingroup ToM. Our findings showed that only outgroup ToM predicted participants’ attribution about intergroup-related factors including discrimination, prejudice, and refugee status/war. This suggests that participants who were better at understanding their refugee peers were more likely to understand the underlying reasons of the intergroup bullying and were more likely to consider these reasons while evaluating the bullying act (H3 was partially supported). Investigating outgroup ToM while examining judgments and reasoning in intergroup social conflicts is important as mental state understanding does not take place automatically and one might need motivation to engage in cognitive resources to understand the mental states of individuals (Carpenter et al., 2016). In other words, contextual factors, including the characteristics of individuals (e.g., being an ingroup or outgroup member) can act as a trigger with which individuals would be willing to use their cognitive resources to understand how others think. However, most of the ToM tasks do not account for the characteristics of the target. Thus, considering the context in designing ToM tasks is especially important when investigating the relation between ToM and intergroup relations.

Furthermore, this study underlines the point that both ingroup and outgroup advanced ToM contribute to participants’ attribution to multiple considerations in their reasoning about the acceptability of intergroup bullying (H5 was supported). Extensive research evidence drawing from the SRD approach demonstrates that socio-cognitive abilities (e.g., ToM) and group processes (e.g., group membership, loyalty to the group, etc.) simultaneously influence the reasoning about social conflicts in intergroup context (Rutland and Killen, 2015). In intergroup contexts, individuals may be drawn to consider group distinctions as they make evaluations, drawing on our cognitive tendency to promote our ingroup and to identify with others who share our racial/ethnic and national ingroup identity (Sani and Bennett, 2004; Davoodi et al., 2020; Feeney et al., 2020). Such complex social situations might lead children and adolescents to weigh multiple considerations in their reasoning, attending to both their identity as well as their moral principles, for instance. Our results provide novel insight by documenting that the more ToM (both ingroup and outgroup) the more likelihood of participants’ referencing more than one category in their reasoning judgments This indicates the possible relationship between ToM and sophisticated reasoning.

Consistent with our prediction, participants’ acceptability judgments were related to their reasoning. More specifically, the lower the participants’ acceptability judgments, the more likely it was that they reasoned about the bullying by referencing fairness, refugee status, discrimination, and harm. For example, participants who evaluated intergroup bullying as less acceptable were more likely to justify these evaluations by giving explanations like “We shouldn’t treat her like this just because she is a refugee from Syrian; It’s racist and discriminatory; It is not fair to bully anyone for any reason.” However, participants’ acceptability judgments were not found to be related to their attribution to social-conventional domain reasoning (prescriptive norms, group functioning) (H4 was partially supported).

Our hypothesis regarding the association between ToM and reasoning about the acceptability of group support to intergroup bullying was not supported. Neither ingroup nor outgroup ToM was significantly related to participants’ reasoning about group support of intergroup bullying of refugee peers. This might be related to the nature of ToM task that we used. More specifically, although we contextualized Strange Stories in terms of our targeted ingroup and outgroup, the stories require participants to attribute mental states to individual characters (either Turkish or Syrian individuals). However, the ToM stories did not involve any group-related process. It is likely that understanding group perspective and group dynamics can be different from the understanding of single-person perspective. Although to our knowledge no ToM task has been developed to evaluate the ability to understand group perspective, previously The Developmental Subjective Group Dynamics (DSGD) model has addressed the importance of recognizing possible differences in groups’ perceptions of the same person (Abrams et al., 2003, 2008). This model has proposed the concept of “theory of social mind (ToSM)” that is particularly related to the ability to differentiate between someone’s own evaluations from peers’ reaction to deviant members of the group. A further interesting avenue for future research could be adapting or developing such measures to understand group perspectives to social conflicts in intergroup contexts. This can help us to understand better how social-cognitive factors might play a role in making judgments about group behavior and awareness of different perspectives in group settings. With regard to the association between participants’ acceptability judgments of group support and reasoning, our results documented that the more participants evaluated group support of intergroup bullying as unacceptable, the more they reasoned about harm. For example, they were more likely to use justifications like “The girl (*Syrian peer*) is already sad. And if they (*group members*) laugh too, she can get more upset.”

### Limitations and Future Directions

Our results should be considered in light of some limitations. First, this study exclusively investigated how adolescents evaluated and reasoned about acts of intergroup bullying of refugee peers and group support of bullying of refugee peers through hypothetical scenarios. Further, we only measured participants’ evaluations and reasoning about one type of bullying (shouting rude words). However, different types of bullying (physical, social exclusion, name-calling) might elicit different evaluations and reasoning. We also did not measure participants’ own experiences as bystanders, bullies, and victims in the context of intergroup bullying. Thus, future research can test whether the current findings can be observed in actual behavior and whether their own experiences in different roles can be related to their evaluations and reasoning. For instance, it may be that observational data collection can clarify exactly what types of contact Turkish and Syrian peers have and whether that intergroup contact is high quality and positive or not. Second, although text-based assessments (e.g., the Strange Stories) provide evidence for ecological validity, it is still likely that real social interactions involve more complex situations that require understanding the perspective of the characters in context. For example, we used the school context to ensure it would make sense to the participants in terms of a common intergroup bullying context, but our ToM measure did not involve such social conflicts scenarios in the school context. Recently, online ToM (e.g., VAMA) tasks have been created to measure advanced mental state understanding that can be applied to different social settings to provide a more naturalistic environment which in turn leads to an increase in ecological validity (Canty et al., 2017; Grainger et al., 2020). Such tasks would be useful to measure ToM abilities in intergroup contexts that involve social conflicts. Further, as discussed earlier, such tasks can be also helpful to measure children’s and adolescents’ simultaneous recognition of possible differences between group perspective and single-person perspective. Further, multi-item larger batteries that capture different domains of ToM can be helpful to understand the relationship between adolescents’ mental state understanding and their reasoning about different types of bullying and social conflicts (Wellman, 2018). Third, extant literature provides evidence for several other factors that can be related to both ToM and bystanders’ reasoning including executive functions (Doenyas et al., 2018; Jenkins et al., 2018; Hoyo et al., 2019; Baker et al., 2021) and empathy (Barchia and Bussey, 2011; Gönültaş et al., 2019). For example, studies showed that executive functions might help individuals to show advanced social reasoning skills, such as those necessary for complex interactions involving moral issues (Doenyas et al., 2018; Baker et al., 2021). Thus, future studies should consider examining other possible factors that might help to understand possible mechanisms between ToM and bystanders’ judgments and reasoning. Fourth, we used different stories to measure ingroup and outgroup ToM considering within-subject design. However, we did not counterbalance the stories across outgroup and ingroup ToM. Although previous studies did not show mean differences in participants’ performance across stories in generalized contexts (Gönültaş et al., 2020), it would be more comparative and informative to counterbalance stories while using them in the context of intergroup. Fifth, the extant literature provides evidence for several other intergroup factors (e.g., prejudice, discrimination, threat perception) that may be related to both bystander judgments and responses (e.g., Gönültaş and Mulvey, 2021a) and Theory of Mind (Gönültaş et al., 2020). However, in the current study, we have only focused on the possible role of group membership (refugee/non-refugee). Future studies should examine how intergroup attitudes, threat perception, social identity, perceived similarity with the targeted outgroup might be related to their reasoning both directly and indirectly (through ToM). Lastly, peer group norms about bullying and Syrian refugee peers can be also related to participants’ reasoning about intergroup bullying (Jones et al., 2012; Gönültaş and Mulvey, 2021a). For example, if adolescents are more likely to be surrounded by peers who do not support bullying and do not have negative attitudes toward Syrian refugees, they might be more likely to evaluate bullying as unacceptable and more likely to detect the discriminatory and prejudicial nature of the intergroup bullying.

## Conclusion

Overall, the findings extend earlier research by examining both ingroup and outgroup ToM in relation to participants’ reasoning to acceptability judgments of intergroup bullying and group support. Understanding the perspective of others who are involved in bullying can be an effective tool to recognize the complex nature of bullying and the underlying reasons behind it especially when it is rooted in prejudice and discrimination. Thus, understanding the perspective of children and adolescents who observe bullying and how they reason about bullying in an intergroup context is an important first step in identifying the mechanism to promote prosocial bystander reactions. Thus, the findings of the current study provide implications for understanding how ingroup and outgroup ToM skills might be related to reasoning about intergroup bullying. This is especially important for intervention programs that tackle intergroup bullying by promoting bystanders’ social cognitive skills.

## Data Availability Statement

The original contributions presented in the study are included in the article/Supplementary Material, further inquiries can be directed to the corresponding author. The data can be shared by the corresponding author, upon reasonable request.

## Ethics Statement

The studies involving human participants were reviewed and approved by North Carolina State University. Written informed consent to participate in this study was provided by the participants’ legal guardian/next of kin.

## Author Contributions

SG made substantial contributions to the design of the project, acquisition of data, analysis, and interpretation of data, and drafting of the manuscript. KM made substantial contributions to the design of the project, analysis, and interpretation of data and revising the manuscript critically for important intellectual content. Both authors contributed to the article and approved the submitted version.

## Conflict of Interest

The authors declare that the research was conducted in the absence of any commercial or financial relationships that could be construed as a potential conflict of interest.

## Publisher’s Note

All claims expressed in this article are solely those of the authors and do not necessarily represent those of their affiliated organizations, or those of the publisher, the editors and the reviewers. Any product that may be evaluated in this article, or claim that may be made by its manufacturer, is not guaranteed or endorsed by the publisher.
