# Supplementary material for: Theory of Mind as a Correlate of Bystanders’ Reasoning About Intergroup Bullying of Syrian Refugee Youth
Source: Front Psychol. 2022 Mar 30;13:815639. doi: 10.3389/fpsyg.2022.815639 (PMC9005638; doi:10.3389/fpsyg.2022.815639)
Supplement: Supplementary file 1 [file Table_1.docx]

**Supplementary Documents**

**Ingroup ToM Stories and Coding**

1- Sedef was born and raised in İstanbul (Turkey). Sedef waited all year for her birthday, because she knew at her birthday she could ask her parents for a rabbit. Sedef wanted a rabbit more than anything in the world. At last her birthday arrived, and Sedef ran to unwrap the big box her parents had given her. She felt sure it would contain a little rabbit in a cage. But when she opened it, with all the family standing round, she found her present was just a boring old set of encyclopedias, which Sedef did not want at all! Still, when Sedef’s parents asked her how she liked her birthday present, she said, ‘‘It’s lovely, thank you. It’s just what I wanted.’’

**Question:** Why did Sedef say this?

**Coding**

**2 points**—reference to white lie or wanting to spare their feelings; some implication that this is for the parents’ benefit rather than just for her, desire to avoid insult

**1 point**—reference to her traits (she’s a nice girl) or relationship (she likes her parents); purely motivational (so they won’t shout at her) with no reference to the parents’ thoughts or feelings; incomplete explanation (she’s lying, she’s pretending)

**0 points**—reference to irrelevant or incorrect facts ⁄ feelings (she likes the present, she wants to trick them)

2- Mrs. Sevinç was born in İstanbul and she is living there for 60 years. Late one night, Mrs. Sevinç is walking home in İstanbul (Turkey). She doesn’t like walking home alone in the dark because she is always afraid that someone will attack her and rob her. She really is a very nervous person! Suddenly, out of the shadows comes a man. He wants to ask Mrs. Sevinç what time it is, so he walks toward her. When Mrs. Sevinç sees the man coming toward her, she starts to tremble and says, ‘‘Take my purse, just don’t hurt me please!’’

**Question:** Why did Mrs. Sevinç say that?

**Coding**

**2 points**—reference to her belief that he was going to mug her or her ignorance of his real intention

**1 point**—reference to her traits (she’s nervous) or state (she’s scared) or intention (so he wouldn’t hurt her) without suggestion that fear was unnecessary

**0 points**—factually incorrect ⁄ irrelevant answers; reference to the man actually intending to attack her

**Outgroup ToM Stories and Coding**

1- Both Uncle Abdul and Karam were born and raised in Syria. One day Uncle Abdul came to visit Karam. Now Karam loves his uncle very much, but today he is wearing a new hat; a new hat which Karam thinks is very ugly indeed. Karam thinks his uncle looks silly in it, and much nicer in his old hat. But when Uncle Abdul asks Karam, ‘‘How do you like my new hat?,’’ Karam says, ‘‘Oh, it’s very nice.’’

**Question:** Why does Karam say that?

**Coding**

**2 points**—reference to white lie or wanting to spare his feelings; some implication that this is for the aunt’s benefit rather than just for his, desire to avoid rudeness or insult

**1 point**—reference to his traits (he’s a nice boy) or relationship (he likes his uncle); purely motivational (so he won’t shout at him) with no reference to the uncle’s thoughts or feelings; incomplete explanation (he’s lying, he’s pretending).

**0 points**—reference to irrelevant or incorrect facts ⁄ feelings (he likes the hat, he wants to trick her)

2- Bahira was born and raised in Şam (Syria). Bahira is always hungry. Today at school it is her favourite meal—meatballs. She is a very greedy girl, and she would like to have more meatballs than anybody else, even though her mother will have made her a lovely meal when she gets home! But everyone is allowed four meatballs and no more. When it is Bahira’s turn to be served, she says, ‘‘Oh, please can I have eight meatballs, because I won’t be having any dinner when I get home!’’

**Question:** Why does Bahira say this?

**Coding**

**2 points**—reference to the fact that she’s trying to elicit sympathy, being deceptive

**1 point**—reference to her state (greedy), the outcome (to get more meatballs) or factual

**0 points**—reference to a motivation that misses the point of sympathy elicitation⁄ deception, or factually incorrect
